# The Amelioration of Hepatic Steatosis by Thyroid Hormone Receptor Agonists Is Insufficient to Restore Insulin Sensitivity in Ob/Ob Mice

**DOI:** 10.1371/journal.pone.0122987

**Published:** 2015-04-07

**Authors:** Alexandro J. Martagón, Jean Z. Lin, Stephanie L. Cimini, Paul Webb, Kevin J. Phillips

**Affiliations:** 1 Diabetes and Metabolic Disease Program, Houston Methodist Research Institute, Houston, Texas, United States of America; 2 Escuela de Biotecnología y Alimentos, Instituto Tecnológico y de Estudios Superiores de Monterrey, Monterrey, NL, Mexico; 3 Center for Nuclear Receptors and Cell Signaling, University of Houston, Houston, Texas, United States of America; University Claude Bernard Lyon 1, FRANCE

## Abstract

Thyroid hormone receptor (TR) agonists have been proposed as therapeutic agents to treat non-alcoholic fatty liver disease (NAFLD) and insulin resistance. We investigated the ability of the TR agonists GC-1 and KB2115 to reduce hepatic steatosis in ob/ob mice. Both compounds markedly reduced hepatic triglyceride levels and ameliorated hepatic steatosis. However, the amelioration of fatty liver was not sufficient to improve insulin sensitivity in these mice and reductions in hepatic triglycerides did not correlate with improvements in insulin sensitivity or glycemic control. Instead, the effects of TR activation on glycemia varied widely and were found to depend upon the time of treatment as well as the compound and dosage used. Lower doses of GC-1 were found to further impair glycemic control, while a higher dose of the same compound resulted in substantially improved glucose tolerance and insulin sensitivity, despite all doses being equally effective at reducing hepatic triglyceride levels. Improvements in glycemic control and insulin sensitivity were observed only in treatments that also increased body temperature, suggesting that the induction of thermogenesis may play a role in mediating these beneficial effects. These data illustrate that the relationship between TR activation and insulin sensitivity is complex and suggests that although TR agonists may have value in treating NAFLD, their effect on insulin sensitivity must also be considered.

## Introduction

Nonalcoholic fatty liver disease (NAFLD) has become the most common chronic liver disease of the developed world, affecting approximately one third of the U.S. population [1]. NAFLD is closely associated with obesity; the majority of patients with NAFLD are obese and approximately half have diabetes [2]. Consequently, the accelerating obesity epidemic has led to a dramatic increase in the number of NAFLD cases worldwide. NAFLD now affects both children and is increasingly being seen in developing countries [3].

NAFLD is used to describe related disorders that arise from a common etiology. While numerous factors, such as defects in mitochondrial β- oxidation, oxidative stress, or ER stress [4] have been implicated in various stages of NAFLD progression, initiation of the disease is caused by a chronic imbalance between triglyceride acquisition, via dietary intake and de novo synthesis, and triglyceride utilization. This imbalance leads to abnormal accumulation of lipid in the liver [3], resulting in hepatic steatosis or fatty liver. While hepatic steatosis is itself benign, under certain conditions or stressors, the excess triglyceride can become lipotoxic, resulting in nonalcoholic steatohepatitis (NASH) [5]. NASH is characterized by inflammation and cellular injury or death of hepatocytes. NASH greatly increases the risk of hepatocellular carcinoma and can lead to fibrosis of the liver, indicating cirrhosis [2].

Approximately 20% of patients with hepatic steatosis will progress to NASH, which increases the risk of liver related mortality by 9–10 fold. NASH is also associated with an increased risk of hepatocellular carcinoma and cardiovascular disease. Once NASH has become cirrhotic, there is a high risk of liver failure, necessitating liver transplantation. The percentage of patients receiving liver transplantation for NAFLD has increased from 0.1% between 1995 to 2000 to approximately 7% currently [6]. Still, following liver transplantation, recurrence of steatosis is common in 60–100% of transplanted patients [7,8].

Despite the increasing burden of NAFLD diagnoses, there are no drugs yet approved for the treatment of hepatic steatosis or NASH. Currently, clinicians have few therapeutic options other than treating the comorbidities of metabolic syndrome that tend to accompany NAFLD [9] and suggesting lifestyle and dietary modifications. Isoform selective agonists of the thyroid hormone receptors (TRs) have been developed primarily for the indication of hypercholesterolemia [10]. However, given the anti-obesogenic actions of TR activation, their use has been proposed for the treatment of other metabolic disorders, including NAFLD. We recently reported that TR agonists reduce serum lipids in a mouse model of severe hyperlipidemia [11]. During these studies we also observed that TR agonist treatment markedly improved hepatic steatosis in these mice; an observation in accordance with other reports indicating that TR agonists can ameliorate fatty liver and NASH in various rodent models of these disorders [12,13].

Here, we test the ability of two TR agonists, GC-1 and KB2115, for their ability to reduce hepatic steatosis in ob/ob mice. Since TR activation has also been associated with reduced insulin sensitivity [14], we additionally investigated the effects of these agonists and T_3_ on insulin sensitivity and glycemic control. While all agonist treatments were found to be highly effective at reducing the burden of steatosis, the effects on insulin sensitivity varied widely, with most treatment conditions leading to impaired glycemic control. Thus, TR agonists dissociate insulin sensitivity from fatty liver, illustrating that the relationship between TR activation and glycemia is complex and suggesting that the effect of TR agonists on insulin sensitivity must be taken into account when considering the use of these compounds for the treatment of NAFLD.

## Results

### TR activation ameliorates hepatic steatosis in ob/ob mice

**Fig 1:**
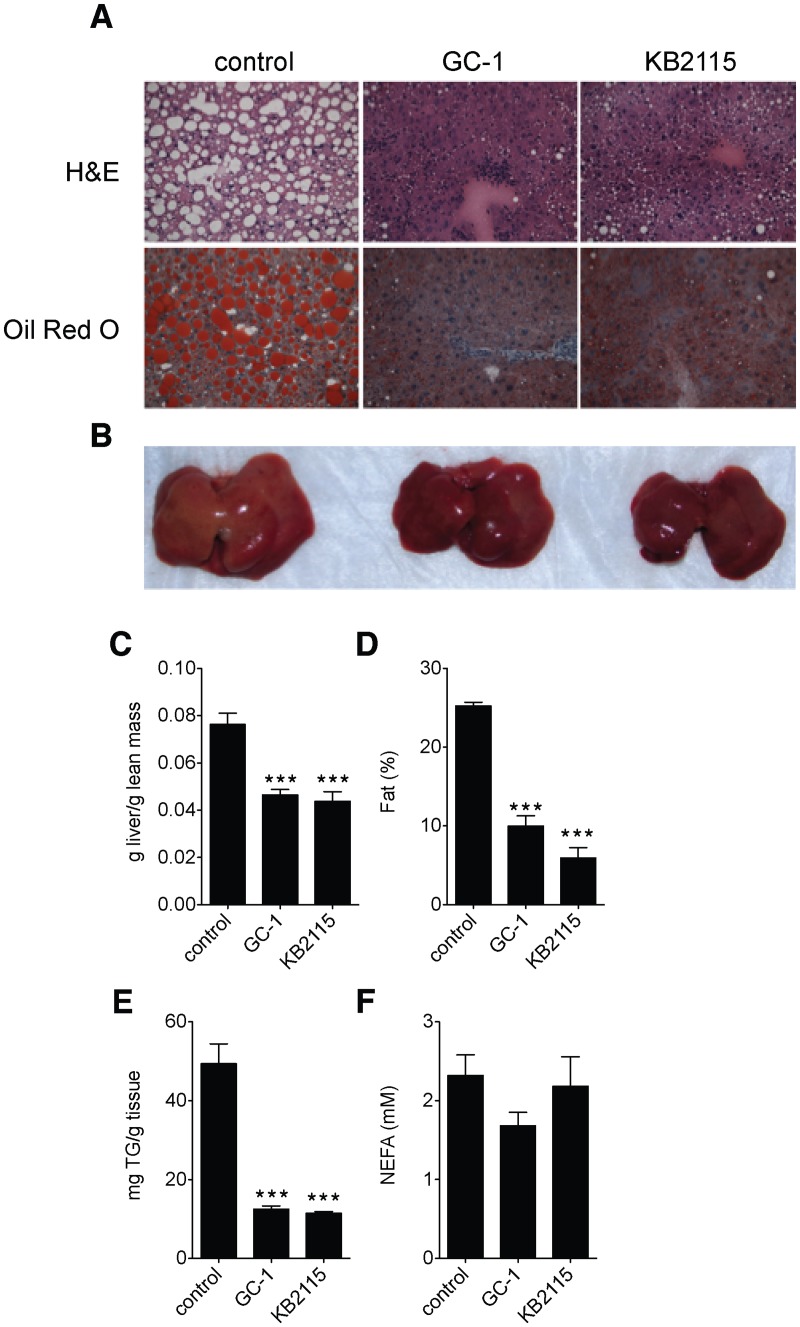
TR agonists ameliorate nonalcoholic fatty liver disease. (A-F) 12-week old ob/ob mice were fed standard chow or chow admixed with GC-1 (0.33 mg/kg-diet) or KB2115 (3.00 mg/kg-diet) for 24 days (*n* = 5–6 per group). (A) Histology analysis of liver steatosis by H&E (top panel) and oil red O (bottom panel) staining from treated and untreated mice. Gross liver images (B) and liver weights (C) taken immediately after extraction. Liver fat composition by qNMR (D) and hepatic triglyceride (E) and NEFA (F) measured from Folch extracts. ***P <0.001. All data are shown as mean ± SEM.

We had observed previously that the TR agonist GC-1 strongly reduced hepatic steatosis in western diet fed LDLR^-/-^ mice (S1 Fig) To further study the relationship between TR activation and fatty liver, we treated ob/ob mice, which develop severe hepatic steatosis, with either GC-1 or the related agonist KB2115. Histological analysis of livers from mice treated with either GC-1 or KB2115 revealed that both compounds elicited a near complete elimination of lipid filled vacuoles that were characteristic of the livers from untreated control mice (Fig 1A). Livers from mice treated with either compound appeared less steatotic upon gross examination (Fig 1B) and weighed substantially less than control mice (Fig 1C). Quantification of steatosis by either NMR of isolated livers or by triglyceride extraction indicated that hepatic triglyceride levels were almost completely normalized following treatment with either agonist (Fig 1D and 1E). Non-esterified fatty acids (NEFA) were unchanged with either agonist (Fig 1F). For comparison, we also tested the endogenous ligand, T_3_, which was also found to substantially reduce hepatic triglyceride levels and ameliorate steatosis (S2 Fig). Thus, TR activation by all agonists tested elicits a marked regression of hepatic steatosis in ob/ob mice.

### Amelioration of hepatic steatosis does not improve insulin sensitivity in ob/ob mice

**Fig 2:**
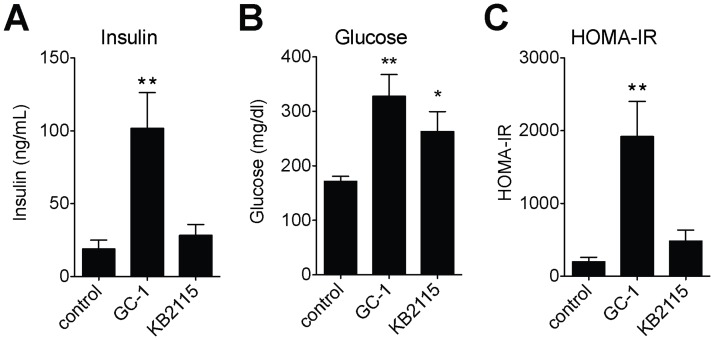
Amelioration of hepatic steatosis does not improve insulin sensitivity in ob/ob mice. Fasting insulin (A), glucose (B) and its derived insulin resistance index (HOMA-IR) (C) from ob/ob mice treated with TR agonists, GC-1 (0.33 mg/kg-diet) or KB2115 (3.0 mg/kg-diet) for 23 days (*n* = 5–6 per group). **P <0.01, *P <0.05. All data are shown as mean ± SEM.

Although the association is complex, there is a clear relationship between obesity, hepatic steatosis, and insulin resistance [15]. To determine whether the reductions in hepatic steatosis that were observed with GC-1 and KB2115 treatment coincided with improvements in insulin sensitivity, we measured fasting glucose and insulin levels and determined insulin sensitivity using the homeostasis model assessment of insulin resistance (HOMA-IR) on mice treated with either GC-1 or KB2115. Despite notably decreasing hepatic steatosis, GC-1 increased serum insulin levels over 5-fold (Fig 2B). Despite increased serum insulin, plasma glucose levels also increased nearly 2-fold (Fig 2A), indicating that the agonist increased insulin resistance nearly 10-fold, as assessed by HOMA-IR (Fig 2C). KB2115 treated mice also exhibited increased fasting glucose, although KB2115 did not increase fasting insulin levels. Increased blood glucose in the face of unchanged insulin levels suggests that insulin sensitivity is also reduced in KB2115 treated animals, although this change did not meet significance criteria (*P* = 0.11, HOMA-IR). These data indicate that the clearance of hepatic steatosis by TR agonists is not sufficient to restore insulin sensitivity in ob/ob mice.

### Effects of TR activation on glycemia are time and dose-dependent

It has long been clinically appreciated that thyroid hormone excess can evoke hyperglycemia and insulin resistance [14,16]. Thus, it should perhaps not have come as a surprise that TR activation by GC-1 and KB2115 could aggravate the hyperglycemia of ob/ob mice. However, we were perplexed by other studies in our group that seemed to indicate that GC-1 could significantly improve glycemic control in multiple mouse models, including ob/ob mice (data not shown). Furthermore, an earlier study involving an agonist closely related to GC-1 (KB141) found that the compound had strong anti-diabetic actions in ob/ob mice [17]. Analysis of the available data led us to suspect that dosage and duration of treatment may be responsible for the seemingly contradictory observations relating TR activation to glycemic control. In order to explore the basis for these discrepancies, we treated mice with either KB2115 or varying doses of GC-1 (referred to here as low-, medium-, and high-dose GC-1) and monitored changes in ad libitum (fed) blood glucose levels during the course of treatment.

**Fig 3:**
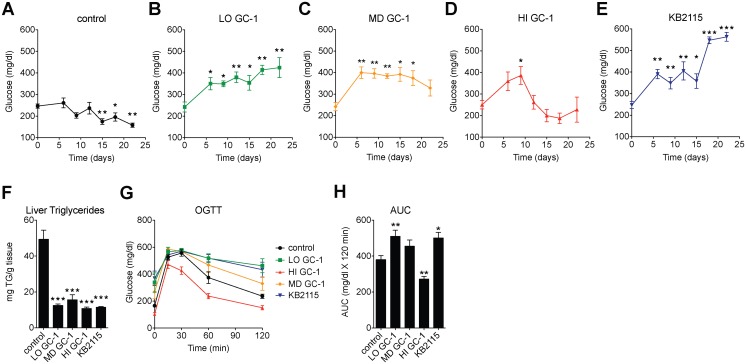
Effects of TR activation on glycemia are time and dose-dependent. (A-H) 12-week old ob/ob mice were treated with varying does of GC-1 (LO, 0.33 mg/kg-diet; MD, 1 mg/kg-diet; HI, 3 mg/kg-diet) and KB2115 (3 mg/kg-diet) (*n* = 5–7 per group). Ad libitum glucose levels at indicated time points (A-E). Hepatic triglycerides levels (F) of mice after 23 days of TR agonist treatment. After 14 days of treatment, an oral glucose tolerance test was performed to assess glycemic control, blood glucose concentrations were measured at indicated time points (G) and the area under the curve (AUC) quantified (**H**). ***P <0.001, **P <0.01, *P <0.05. All data are shown as mean ± SEM.

As demonstrated in Fig 3A–3E, changes in serum glucose were highly dependent on dose and time of treatment. Glucose levels of untreated control mice were relatively stable, exhibiting a slight but significant decrease over time (Fig 3A). The drop in ad libitum glucose levels as ob/ob mice age has been observed previously and is attributed to increased insulin production that results from β-cell hyperplasia [18,19]. In comparison, glucose levels of all treatment groups were significantly elevated after 9 days of treatment (Fig 3B–3E). Glucose levels of mice treated with low-dose GC-1 or KB2115 continued to increase over time, leading to pronounced hyperglycemia by the end of the study (Fig 3B and 3E). In contrast, the trajectory of glucose values for mice treated with the highest dose of GC-1 was quite distinct from that of KB2115 and low dose GC-1. Following an initial increase in hyperglycemia, glucose levels reversed course and declined rapidly after the ninth day of treatment. By the end of the study, glucose levels of this group were not significantly different from the starting values and were not significantly different from untreated control mice (Fig 3A and 3D). Glucose levels of mice treated with medium-dose GC-1 followed a course that was intermediate between that seen with low- and high-dose GC-1.

To further examine how the various treatments affected glycemic control, we measured oral glucose tolerance in mice treated similarly as in Fig 3. The regression of hepatic steatosis was similar in all treatment groups (Fig 3F). KB2115, low- and medium-dose GC-1 decreased glycemic control and reduced glucose disposal, although the impairment was not as large in mice receiving an intermediate dose of GC-1 as it was with the lower dose of GC-1 or with KB2115 (Fig 3G and 3H). While all other treatments led to impaired glycemic control, the highest dose of GC-1 was found to improve glycemic control and increase glucose disposal. These data demonstrate that the effects of TR agonists on glycemia are highly dependent upon dosage used and that responses to TR agonism can vary widely, ranging from substantial improvement to considerable impairment of glycemic control.

**Fig 4:**
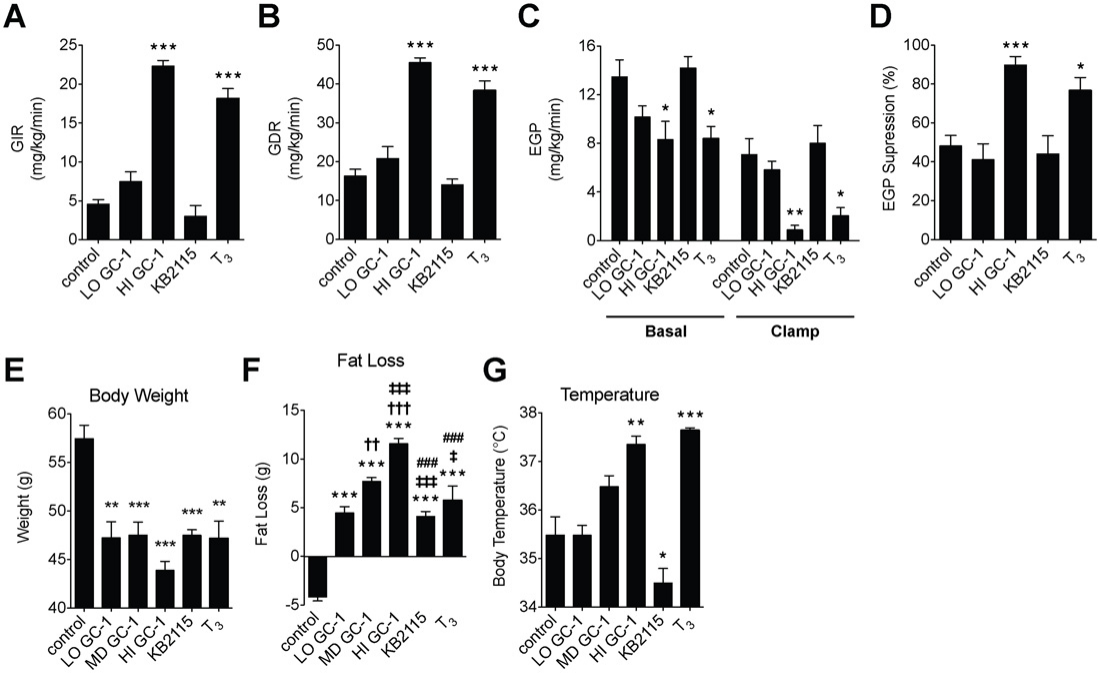
Improvement in glycemic control is due to increase insulin sensitivity. (A-D) Hyperinsulinemic-euglycemic clamps were performed on 12-week old ob/ob mice treated with GC-1 (LO, 0.03 mg/kg; HI, 0.3 mg/kg), KB2115 (0.3 mg/kg) and T_3_ (0.06 mg/kg) via daily ip injection. Glucose infusion rate (A), glucose disposal rate (B) endogenous glucose production (C) and EGP supperion (D) were measure in mice after 10 days of treatment. Body weight (E), fat loss (F) and body temperature (G) of mice after 23 days of treatment.***P <0.001, **P <0.01, *P <0.05 compared with control; ††† *P* <0.001, †† *P* <0.01 compared with LO GC-1; ‡‡‡ *P* <0.001 compared with MD GC-1; ### *P* <0.001 compared with HI GC-1. All data are shown as mean ± SEM.

To obtain a more quantitative measure the effects of the TR agonists on insulin sensitivity, we performed hyperinsulinemic-euglycemic clamps on ob/ob mice treated with low-dose GC-1, high-dose GC-1, KB2115, or vehicle. Additionally, in order to compare the effects of the synthetic TR agonists to that of the endogenous hormone, we also treated mice with a pharmacological dose of T_3_. Insulin sensitivity, as measured by glucose infusion rate was markedly improved in mice treated with high-dose GC-1 or T_3_, while low-dose GC-1 and KB2115 had no effect on insulin sensitivity (Fig 4A). Glucose disposal rate, which represents the glucose uptake of extra-hepatic tissues, also increased substantially in mice treated with high-dose GC-1 or T_3_, yet was unchanged with low-dose GC-1 or KB2115 treatment (Fig 4B). In a similar fashion, endogenous glucose production (EGP) in both basal and insulin stimulated states, was decreased with high-dose GC-1 or T_3_ treatment, yet unaltered with low-dose GC-1 or KB2115 and insulin stimulated suppression of EGP increased only following high-dose GC-1 or T_3_ treatment (Fig 4C and 4D). These data indicate that, despite the ability of low-dose GC-1 and KB2115 to ameliorate hepatic steatosis (Fig 1), neither treatment elicits improvements in whole body, peripheral, or hepatic insulin sensitivity. Conversely higher doses of GC-1 and supraphysiological doses of T_3_ can markedly improve insulin sensitivity in ob/ob mice both by improving peripheral extra-hepatic glucose disposal and by increasing hepatic insulin action. These results further demonstrate that changes in insulin sensitivity elicited by TR agonists are highly dependent on the compound being utilized and dosage.

### Improvements in glycemic control coincide with significant increases in body temperature

All TR agonist treatments led to significant weight and fat loss (Fig 4E and 4F). Similar weight loss was observed with all treatments except for the highest dose of GC-1, which elicited greater weight loss than the other treatments. All treatments also elicited fat loss, with higher doses of GC-1 resulting in greater fat loss. Thus, there appears to be little correspondence between weight loss brought about by the various TR agonists and insulin sensitivity. Since metabolic increase brought about by thyroid hormone and TR activation is often attributed to thermogenesis, as a surrogate indicator of potential thermogenesis, we examined how all treatments effected body temperature (Fig 4G). Mice treated with high-dose GC-1 or T_3_ exhibited pronounced elevation of body temperature. Temperature of mice treated with KB2115 was decreased relative to untreated control mice, while body temperature was unchanged with low- and medium-doses of GC-1. Thus, although all compounds and treatments studied markedly reduced the severity of fatty liver (except for T_3_, not determined) and induced weight and fat loss, only those that induced an increase in temperature resulted in improved Insulin sensitivity or glycemic control.

### Impaired glycemia coincides with the induction of glucose-6-phosphatase

**Fig 5:**
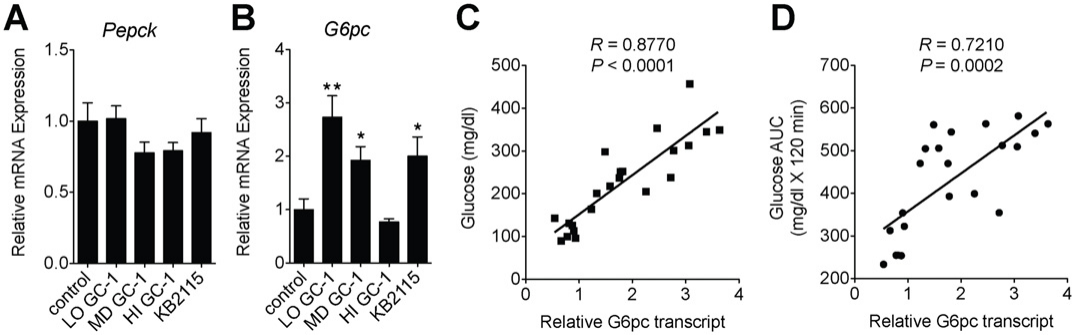
Impaired glycemia coincides with the induction of glucose-6-phosphatase. (A, B) Hepatic gene expression analysis of G6pc and Pepck from ob/ob mice treated with varying does of GC-1 (LO, 0.33 mg/kg-diet; MD, 1 mg/kg-diet; HI, 3 mg/kg-diet) and KB2115 (3 mg/kg-diet) for 23 days (*n* = 5–6 per group). Correlation between hepatic G6Pase expression and fasting glucose (C) and glycemic control (AUC) (D).**P <0.01, *P <0.05. All data are shown as mean ± SEM.

Several reports have implicated TR activation in the induction of hepatic gluconeogenesis [20–22]. Since this action has a clear association with insulin resistance [23], we questioned whether the induction of hepatic gluconeogenesis by GC-1 and KB2115 might contribute to the reduced glycemic control and the failure to improve insulin sensitivity in mice treated with these agonists. We measured changes in the hepatic expression of glucose-6-phosphatase (G6pc) and phosphoenolpyruvate carboxykinase (Pepck), both enzymes involved in key regulatory steps of gluconeogenesis. Unlike other reports investigating the effects of T_3_ on hepatic gene expression [24], we did not observe the induction of PEPCK with any treatment (Fig 5A and S3 Fig), while both GC-1 (low- and medium-dose) and KB2115 significantly increased expression of G6pc (Fig 5B). G6pc expression was unaltered following high-dose GC-1 treatment. Since treatment groups with impaired glucose tolerance seemed to coincide with those in which glucose-6-phosphatase expression was increased, we analyzed the correlation between G6pc expression in agonist treated mice with fasting glucose levels and glycemic control (AUC from Fig 3H). Indeed, G6pc expression in individual mice treated with TR agonists was found to correlate quite strongly with plasma glucose levels as well as glucose tolerance (Fig 5C and 5D). Similar to the results seen with the synthetic agonists, in mice treated with a dose of T_3_ that was sufficient to ameliorate hyperglycemia, Pepck levels were unaltered, while G6pc levels were reduced (S3 Fig).

## Discussion

This study demonstrates clearly that the amelioration of hepatic steatosis by TR agonists is not sufficient to improve insulin sensitivity, at least in ob/ob mice. While it has become apparent that there is not a direct causal relationship linking fatty liver to insulin resistance [25], reductions in hepatic triglyceride levels generally coincide with improved insulin sensitivity. While somewhat exceptional, we are aware of several reports where the ablation of proteins involved in triglyceride synthesis prevented the onset of fatty liver but also coincided with reduced insulin sensitivity or glycemic control. The loss of GPAT1 leads to impaired insulin sensitivity despite preventing hepatic steatosis in ob/ob mice [26,27]. Similarly, ob/ob mice lacking stearoyl-CoA Desaturase-1 do not develop fatty liver, but have impaired glycemic control, nonetheless [28]. That these studies, like this report, involve ob/ob mice may suggest the possibility that this effect is related to the leptin deficiency of these mice. The loss of leptin signaling, in addition to causing extreme obesity, also results in metabolic alterations that include diminished brown fat mediated thermogenesis, rendering ob/ob mice hypothermic. That the only treatments in this study found to improve either insulin sensitivity or glycemic control, high-dose GC-1 or T_3_, were also those that increased body temperature and restored euthermia to ob/ob mice does suggest to us that the restoration of thermogenesis may be an important component of the improved insulin sensitivity in these mice. Thus, the results here suggesting a relationship between improvements in insulin sensitivity and thermogenesis may be particular to these mice. However, the ability of TR agonists to dissociate the amelioration of fatty liver and improvements in insulin sensitivity appears to be a general phenomenon, as similar results were observed in high-fat fed rats [29]. In that study, much like the current findings, it was shown that while both GC-1 and KB2115 reduced hepatic steatosis substantially, neither compound elicited improvements in insulin sensitivity.

That the effects of TR agonism on glycemia can vary widely and are time-, dose-, and agonist-dependent attests to the complex relationship between thyroid hormone signaling and insulin sensitivity. Indeed, it is not clear from literature reports relating TR action to insulin sensitivity whether TR agonism is pro- or anti-diabetic. In humans, it’s generally accepted that thyroid hormone signaling decreases insulin sensitivity and increases serum glucose levels [14]. In patients with type II diabetes, hyperthyroidism typically leads to further impaired glycemic control. However thyroid hormone has also been reported to ameliorate hyperglycemia and hyperinsulinemia when administered to a severely insulin resistant patient [30]. In rodent models of insulin resistance, the TR agonist KB141 was shown to elicit striking improvements in glycemic control and insulin sensitivity when administered to ob/ob mice [17], while the agonist MB07811 has been reported to reduce fasting glucose levels in diet-induced obese mice [31].

In this study, we find that lower doses of GC-1 impair glycemic control and insulin sensitivity, while higher doses improve both. This suggests to us that multiple, competing mechanisms involved in regulating glycemia are being affected by TR agonists. All treatments tested increased glucose levels at early time points (Fig 3B–3E), which we believe indicates that there is a mechanism common to all treatment groups by which TR activation induces hyperglycemia. In mice treated with higher doses of GC-1, glucose levels increase at early time points, only to reverse course and decrease at later time points (Fig 3C and 3D). To us, this indicates that a second mechanism, operative only at higher doses of GC-1 and longer treatment times, mediates reductions in serum glucose. The suggestion that TR activation affects multiple mechanisms that control insulin sensitivity should not be surprising, given the pleiotropic metabolic effects of the TRs. However, the common effects of TR activation, including those observed in this study: weight and fat loss and the amelioration of fatty liver would all be expected to result in improved insulin sensitivity, which prompts the question, “What is responsible for the reduced insulin sensitivity that is often observed with thyroid hormone excess or TR agonist treatment?”

While the answer to this question is not yet clear, there is accumulating evidence that the induction of hepatic gluconeogenesis by TR agonists may be responsible for reductions in insulin sensitivity. A recent study, which also explored the effect of TR agonists on hepatic steatosis, found that GC-1, despite reducing hepatic triglyceride content, reduced whole body insulin sensitivity in rats by increasing hepatic glucose production [29]. Increased gluconeogenesis was attributed to a combination of increased *G6pc* expression and increased glycerol release from adipocytes, which stimulates hepatic gluconeogenesis. Increased hepatic *G6pc* has been shown to be sufficient to elicit insulin resistance [32] and hepatic *G6pc* is rapidly induced by T_3_ *in vivo* [21] and was the most highly induced gene *in vitro* when HepG2 cells were treated with either GC-1 or T_3_ [33], suggesting that *G6pc* induction is a direct effect of TR activation in hepatocytes. In the current study, *G6pc* expression correlates quite well with serum glucose levels and glucose tolerance. This leads us to hypothesize that the liver specific induction of *G6pc* could be responsible for the hyperglycemia that is often observed accompanying TR agonism and may explain why, in this study, treatment with low-dose GC-1 or KB2115 failed to improve hepatic insulin sensitivity (Fig 4D) despite substantially reducing hepatic lipid burden.

If hepatic glucose production is responsible for reduced glycemic control, what beneficial action counteracts this effect and mediates improved glucose tolerance, as observed with the highest dose of GC-1 and T_3_? It is clearly not related to the amelioration of hepatic steatosis. There is a correspondence between those treatments that improve insulin sensitivity and glycemic control and increased body temperature, which suggests to us that the beneficial effects of TR activation may arise as a consequence of thermogenesis. Agonist treatments that did not increase body temperature (KB2115 and the lowest dose of GC-1) produced the most pronounced glucose intolerance (Fig 4B, 4C and 4F) and failed to improve insulin sensitivity. However, treatments that increased body temperature, the highest dose of GC-1 and T_3_, improved insulin sensitivity quite notably. Thyroid hormone and the TRs have a well established association with thermogenesis and metabolic increase that is attributed to their involvement in regulating adaptive thermogenesis in adipose tissues, as well as by increasing obligatory thermogenesis in other tissues [34]. Recent reports suggest that the induction of thermogenesis may directly affect improvements in glycemic control. The induction of adaptive thermogenesis increases glucose disposal and improves glycemic control in obese mice [35,36], and increased brown fat activity resulting from thyroid hormone administration has been reported to improve diabetic control in a patient with severe diabetes [30].

As key metabolic mediators, TRs can modulate metabolism via both central actions [37,38] and effects in peripheral tissues. Thus, TR action in all tissues must be considered when considering changes in whole body metabolism, such as alterations in insulin and glucose levels. Both GC1 and KB2115 are isoform selective agonists, designed to target the predominant TR isoform in the liver, TRβ, thus avoiding deleterious cardiac effects that result from activation of TRα [10], the major TR isoform in the heart. As both GC-1 and KB2115 activate TR target genes in the liver [11], a major tenet of our model (S4 Fig) is that it is hepatic TR activation that is responsible for impaired glycemic control, which implies that TR activation either centrally or in extra-hepatic tissues is necessary to increase insulin sensitivity. While tissue selective activity was originally sought by designing agonists that selectively target TRβ, it is becoming clear that tissue selective uptake by members of the monocarboxylate transporter (MCT) and organic anion transporting polypeptides (OATP) families of membrane transporters is also important for the distribution and tissue selective actions of thyroid hormone and TR agonists [39,40]. Relatedly, KB2115 has been reported to be uptaken in a liver-specific fashion, rendering the compound largely inactive in extra-hepatic tissues [41]. It is this difference in tissue distribution that we propose is to account for the differential systemic effects of TR agonists including KB2115 and GC-1. In this study, multiple doses of KB2115 were not tested, as, unlike GC-1, preliminary studies did not reveal any dose of KB2115 that resulted in improved glycemic control or substantially affected the expression of TR target genes in extra-hepatic tissues (data not shown), supporting the idea that KB2115 is a liver specific compound. It is the liver specificity of KB2115, which renders it unable to produce extra-hepatic actions, such as the induction of thermogenesis, that we suggest is responsible for the compounds' pronounced aggravation of hyperglycemia. Presumably, low doses of GC-1 behave similarly, principally activating hepatic TRs, due to the liver selective tissue distribution profile of the ligand [42]. However, at higher doses, GC-1 begins to also activate TRs in tissues outside the liver, which counteracts the deleterious effects of hepatic TR agonism on glycemia, allowing this compound to potentially improve insulin sensitivity at appropriate doses.

In conclusion, we show that TR agonists can induce a regression of hepatic steatosis, although the effects of these compounds on glycemia are highly variable and are time-, dosage-, and agonist-dependent. These results illustrate why reports linking TR activation to insulin sensitivity diverge widely. As a demonstration of the complex relationship between TR action and insulin sensitivity, we show that the same compound can elicit both positive and adverse outcomes, depending on the dosage. Given the preponderance of hepatic steatosis and NAFLD, there is a great need for new therapeutics to treat these disorders. As TR agonists appear to be highly effective at reducing hepatic triglycerides and ameliorating fatty liver they could potentially be used as therapeutics to treat hepatic steatosis, although their effect on glycemia will also need to be considered.

## Materials and Methods

### Animals and diets

Twelve-week old male ob/ob mice were purchased from Harlan Laboratories and housed in a temperature-controlled environment with 12 h light/dark cycles and fed standard irradiated rodent chow *ad libitum* at Houston Methodist Research Institute. Mice were randomly divided in groups and fed standard 2918 rodent chow (Harlan) or standard chow admixed with GC-1 or KB2115 ad libitum at the doses indicated per figure. Animals were sacrificed by carbon dioxide exposure after 23–25 days of treatment, and tissues were collected and frozen at -80°C. All animal studies were reviewed and approved by Houston Methodist Research Institute Institutional Animal Care and Use Committee (Animal Use Protocol 0312–0017).

### Hyperinsulinemic-euglycemic clamp

A catheter was implanted into the right internal jugular vein before the hyperinsulinemic-euglycemic clamp. After recovery, mice were administered GC-1 (0.03 or 0.3 mg/kg), KB2115 (0.3 mg/kg) or T_3_ (0.06 mg/kg) via intraperitoneal injection for 10 days. On the day of the clamp experiment, conscious, overnight-fasted mice received a primed (10 μCi) and constant rate intravenous infusion (0.1 uCi/min) of [3-^3^H] glucose to measure basal glucose turnover. After 60–75 minutes of labeled glucose infusion, the hyperinsulinemic-euglycemic clamp was performed with continuous infusion of insulin (12 mU/kg/min) and variable infusion of 25% glucose to maintain euglycemia (~120 mg/dl). Blood samples were collected by tail bleeding (approximately every 10 min) to measure blood glucose concentrations. Hyperinsulinemic-euglycemic clamps were performed at the Mouse Metabolism Core at Baylor College of Medicine.

### Histology

Approximately 5 mg of each specimen liver was fixed in 10% buffered formalin phosphate (Fischer Scientific) and embedded in paraffin, sectioned at 5μm, and stained with hematoxylin and eosin by Houston Methodist Pathology Core. For oil red O staining, livers were fixed in 10% buffered formalin phosphate, frozen, sectioned on a cryostat, and stained by Baylor College of Medicine’s Comparative Pathology Laboratory.

### Triglyceride analysis

Each liver was homogenized and total lipids were extracted according to the Folch method (CHCl_3_: MeOH, 2:1)[43], precipitated with 0.6% NaCl, evaporated for dryness in a rotatory vacuum evaporator (Eppendorf), and resuspended in 1% triton X-100 PBS. Triglyceride levels from plasma and liver were assayed with infinity triglyceride colorimetric reagent (Thermo Scientific).

### Body composition analysis

Body fat percentage was obtained using quantitative NMR (EchoMRI).

### Body Temperature

Core body temperature was measured at the end of treatment using a digital thermometer with RET-3 rectal probe for mice.

### Non-esterified fatty acid measurement

Prior to sacrifice, blood was obtained by retro-orbital vein puncture under anesthesia, and collected in heparinized tubes. Plasma was isolated via centrifugation and stored at -80°C until analysis. Serum NEFA quantification was performed using HR series NEFA kit (WAKO).

### Insulin analysis

Prior to sacrifice, blood was collected in heparinized tubes. Plasma was isolated via centrifugation and stored at -80°C until analysis. Plasma insulin levels were determined using a Rat/Mouse Insulin ELISA Kit (Millipore) for rodent plasma.

### HOMA-IR

Homeostatic model assessment for insulin resistance was calculated with the following formula: fasting glucose (mg/dl) x fasting insulin (μU/mL) / 405.

### RNA isolation and quantification of gene expression

Each liver was homogenized and total RNA was isolated using TRIzol reagent (Invitrogen) and RNeasy mini kit (QIAGEN). First strand cDNA was synthesized using SuperScript VILO synthesis kit (Invitrogen). Quantitative RT-PCR (RT-qPCR) was performed using TaqMan gene expression probes in a LightCycler 480 real time PCR system (Roche). Primer information can be provided upon request.

### Oral glucose tolerance test

Mice were fasted for 5 hours, and OGTT was performed. A dose of 2g/kg glucose was given to each mouse by oral gavage and tail vein blood was drawn, and blood glucose was measured with OneTouch UltraMini (LifeScan, Inc.), at 0, 15, 30, 60, and 120 minutes.

### Statistics and replication

With the exception of the hyperinsulinemic-euglycemic clamp study, all experiments in the manuscript were performed at 2–3 times, with similar results obtained for each trial. The number of replicates noted in the figure captions represents results from one experimental trial; data was not pooled from multiple experiments. Comparison between two groups was assessed by un-paired two-tail Student’s *t* test. Correlation between G6pase expression and glucose and AUC was evaluated by Pearson’s correlation coefficient. *P* values less than 0.05 were considered significant. Values are presented as mean ± S.E.M.

## Supporting Information

S1 Fig The TR agonist GC-1 decreases hepatic steatosis in western diet fed LDLR^-/-^ mice. (A, B) LDLR^-/-^ mice fed a western diet containing 0.2% cholesterol were administered GC-1 (4.8 mg/kg-diet) or a control diet (n = 5–6 per group) for 14 days. (A) Hepatic triglyceride levels were measured from Folch extracts and gross liver images (B) were taken immediately after extraction. ***P < 0.001. All data are shown as mean ± SEM.
(TIF)

S2 Fig T_3_ decreases hepatic steatosis in ob/ob mice. (A, B) Male ob/ob were administered T_3_ (0.06 mg/kg) or vehicle via daily intraperitoneal injections for 21 days (n = 4–6). (A) Hepatic triglyceride levels were measured from Folch extracts and liver sections were stained with H&E (B). Scale bar, 100 μm. *P < 0.05. All data are shown as mean ± SEM.
(TIF)

S3 Fig T_3_ does not induce gluconeogenesis. (A, B) Hepatic gene expression of G6pc and Pepck from ob/ob mice treated with T_3_ (0.06 mg/kg) or vehicle via daily intraperitoneal injections for 21 days (n = 4–6).
(TIF)

S4 Fig Proposed mechanistic rationale for the pro- versus anti-diabetic actions of TR agonists. At low doses, both compounds activate TR target genes in the liver due to their selective affinity for TRβ, the predominant TR isoform in the liver. However, at higher doses GC-1 begins to induce genes in extra-hepatic tissues, resulting in the induction of thermogenesis and improvements in insulin sensitivity and glycemic control. In addition to TRβ selectivity, KB2115 has an additional level of tissue selectivity due to selective uptake into the liver, rendering the compound unable to active TR target genes in extra-hepatic tissues, induce thermogenesis, or improve insulin sensitivity.
(TIF)
