# Supplementary figures and images for: The Amelioration of Hepatic Steatosis by Thyroid Hormone Receptor Agonists Is Insufficient to Restore Insulin Sensitivity in Ob/Ob Mice
Source: PLoS One. 2015 Apr 7;10(4):e0122987. doi: 10.1371/journal.pone.0122987 (PMC4388544; doi:10.1371/journal.pone.0122987)

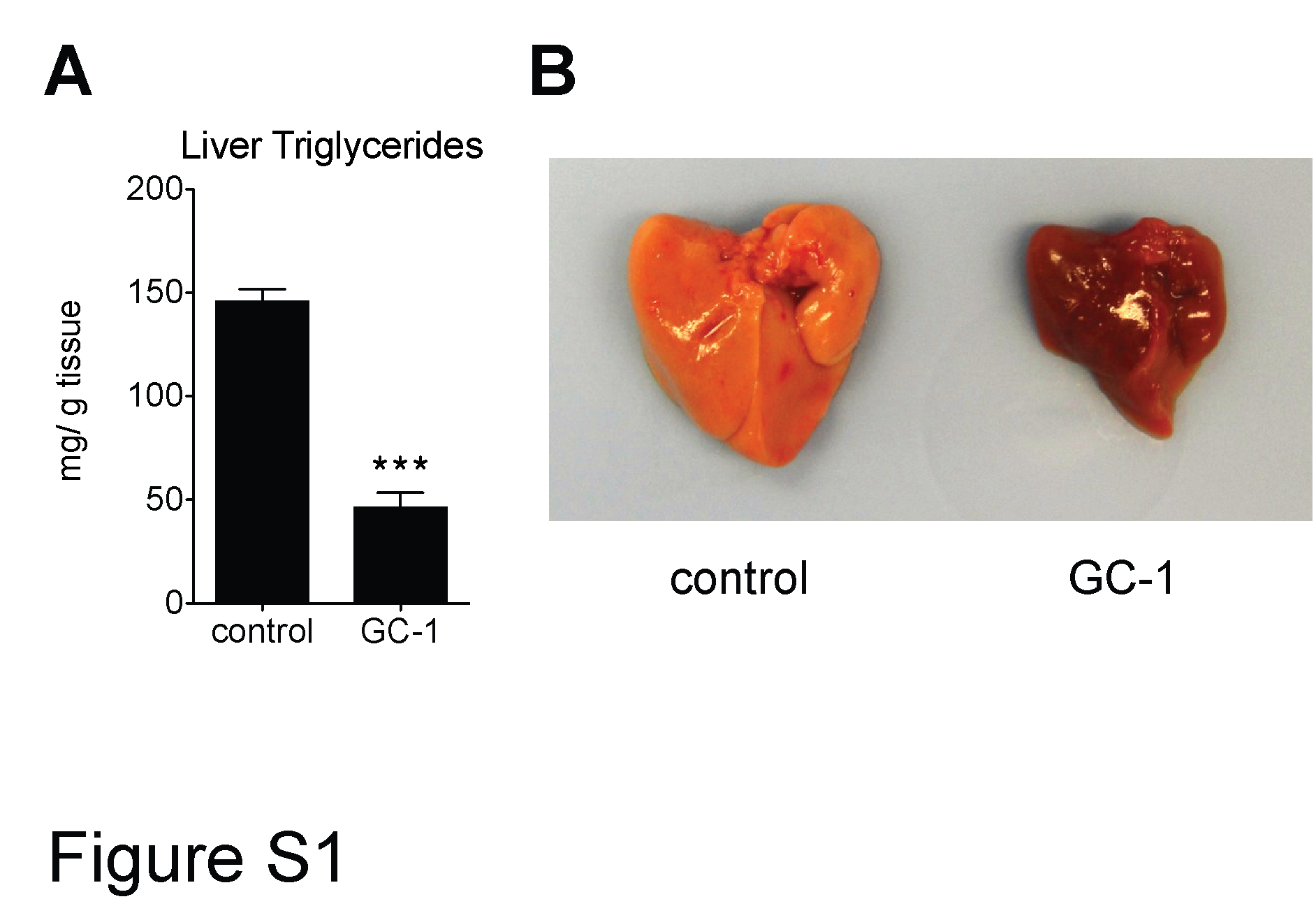

Supplement: S1 Fig — (A, B) LDLR-/- mice fed a western diet containing 0.2% cholesterol were administered GC-1 (4.8 mg/kg-diet) or a control diet (n = 5–6 per group) for 14 days. (A) Hepatic triglyceride levels were measured from Folch extracts and gross liver images (B) were taken immediately after extraction. ***P < 0.001. All data are shown as mean ± SEM. (TIF) [file pone.0122987.s001.tif]

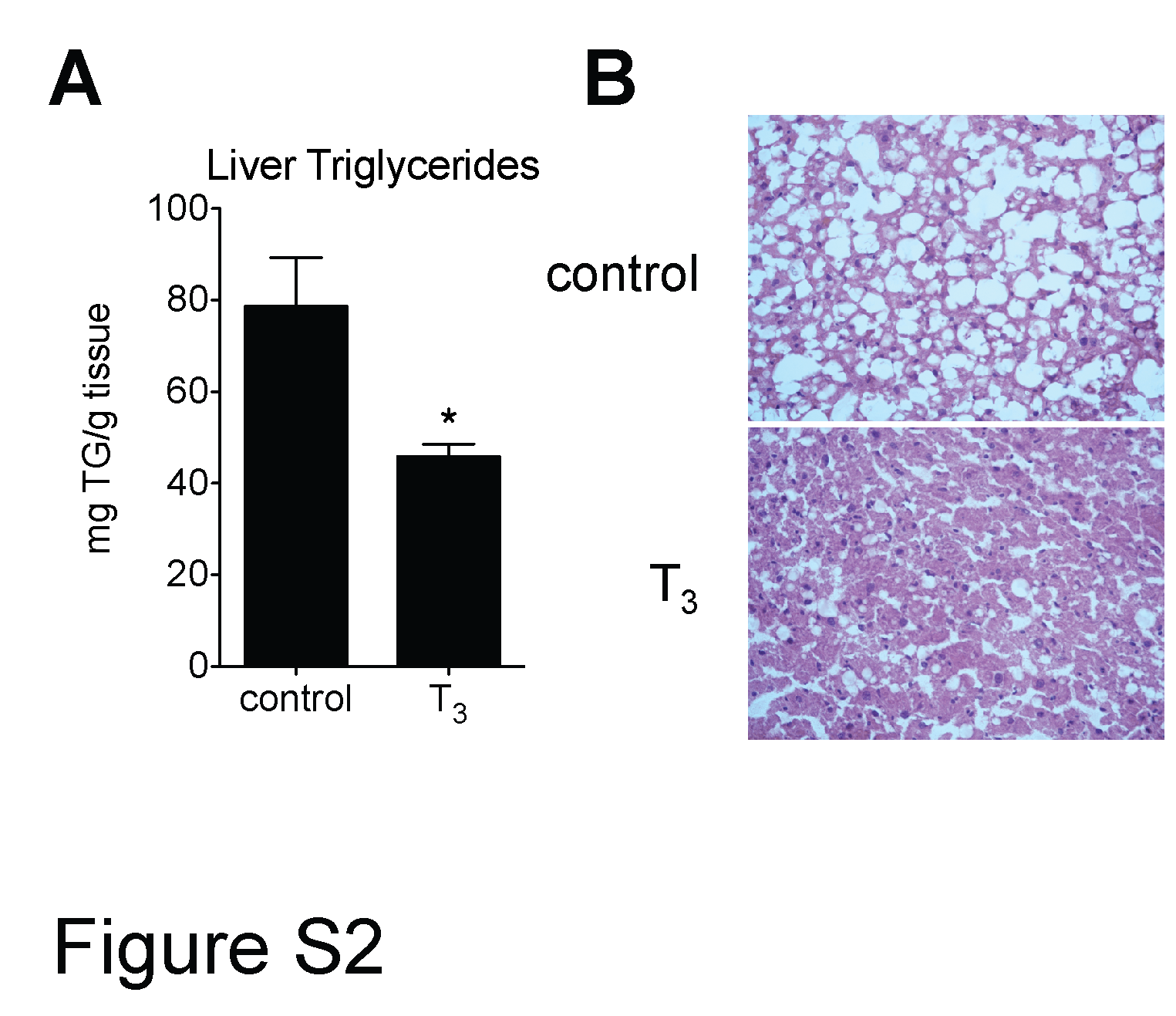

Supplement: S2 Fig — (A, B) Male ob/ob were administered T3 (0.06 mg/kg) or vehicle via daily intraperitoneal injections for 21 days (n = 4–6). (A) Hepatic triglyceride levels were measured from Folch extracts and liver sections were stained with H&E (B). Scale bar, 100 μm. *P < 0.05. All data are shown as mean ± SEM. (TIF) [file pone.0122987.s002.tif]

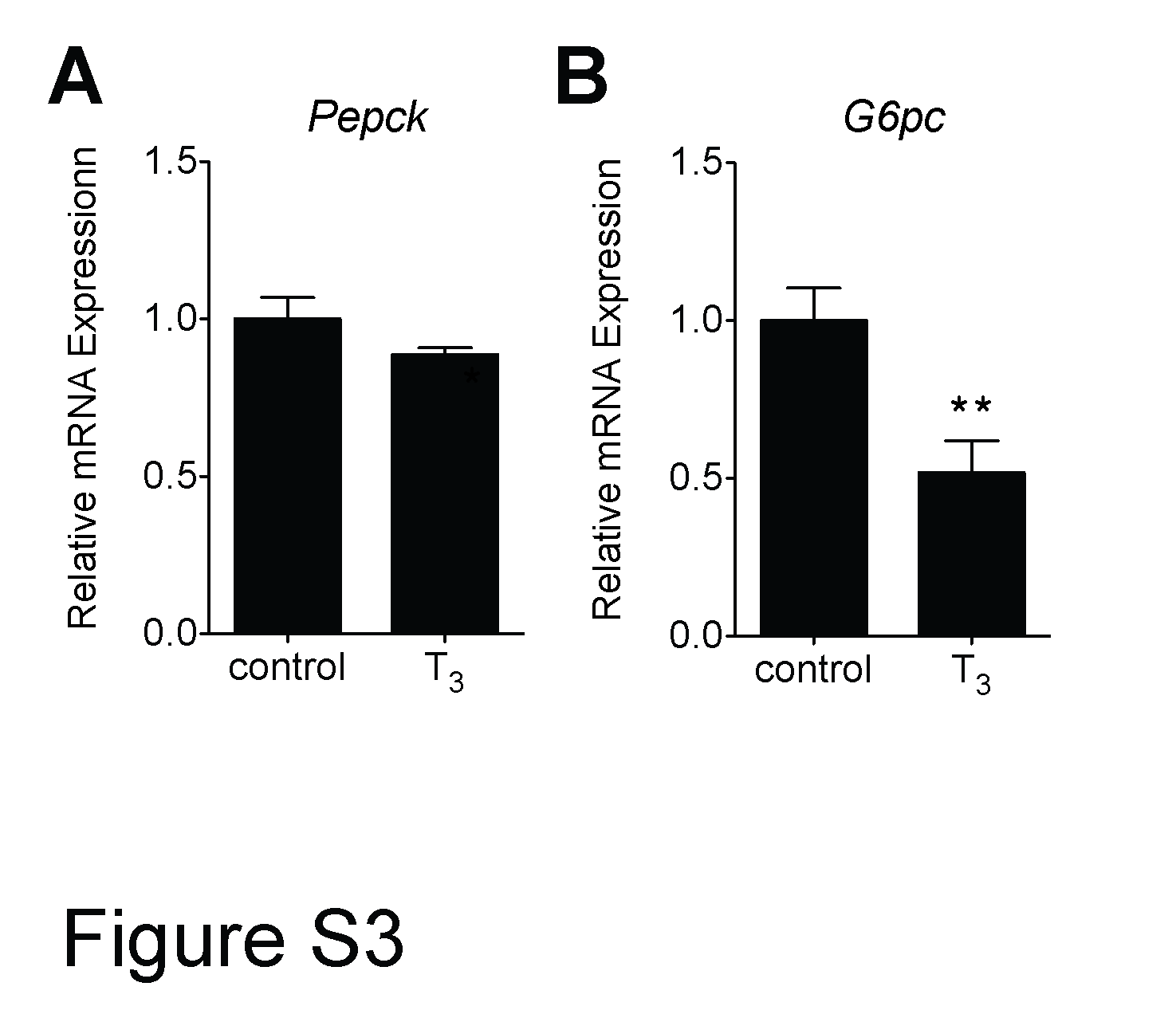

Supplement: S3 Fig — (A, B) Hepatic gene expression of G6pc and Pepck from ob/ob mice treated with T3 (0.06 mg/kg) or vehicle via daily intraperitoneal injections for 21 days (n = 4–6). (TIF) [file pone.0122987.s003.tif]

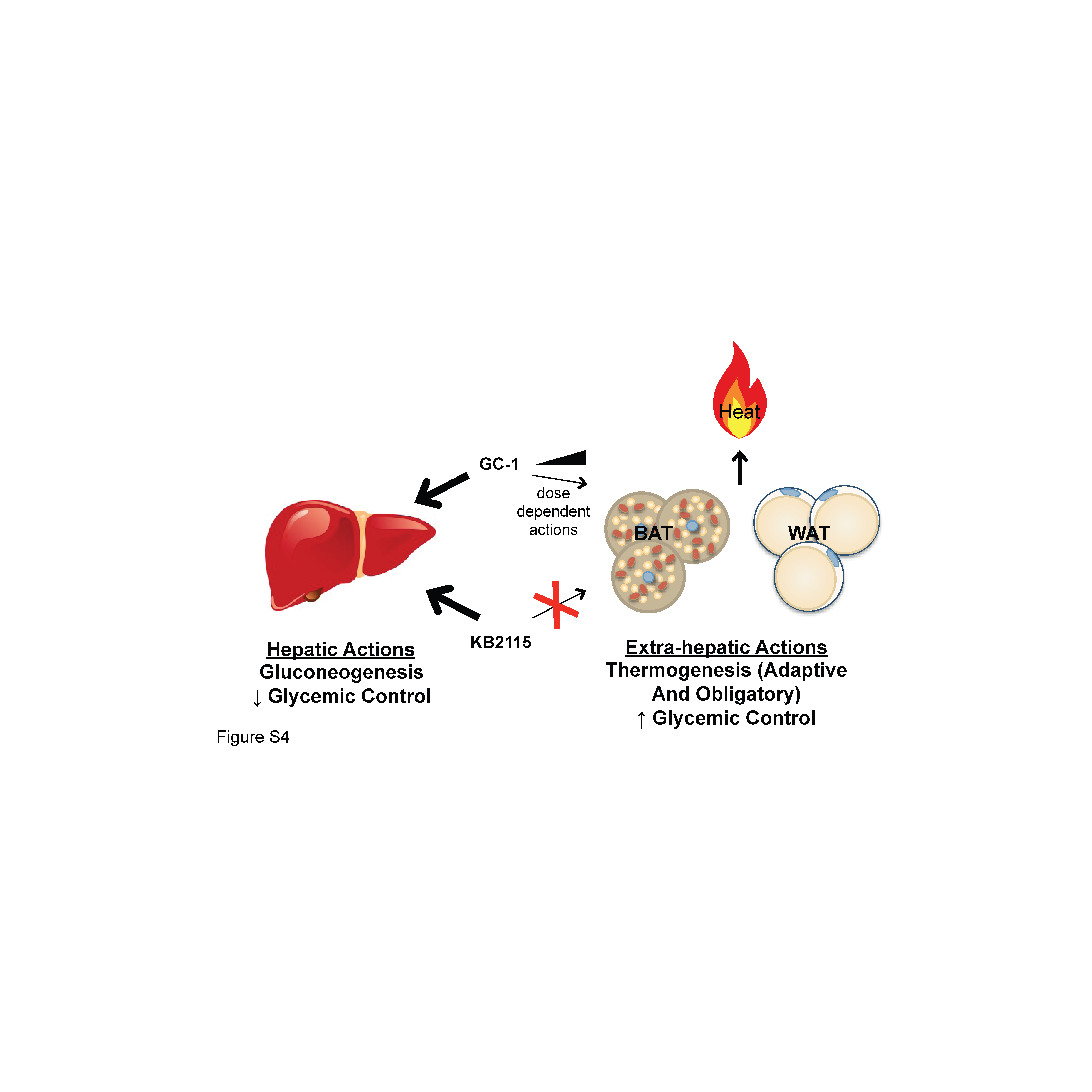

Supplement: S4 Fig — At low doses, both compounds activate TR target genes in the liver due to their selective affinity for TRβ, the predominant TR isoform in the liver. However, at higher doses GC-1 begins to induce genes in extra-hepatic tissues, resulting in the induction of thermogenesis and improvements in insulin sensitivity and glycemic control. In addition to TRβ selectivity, KB2115 has an additional level of tissue selectivity due to selective uptake into the liver, rendering the compound unable to active TR target genes in extra-hepatic tissues, induce thermogenesis, or improve insulin sensitivity. (TIF) [file pone.0122987.s004.tif]
